# F4/80^+^ Kupffer Cell-Derived Oncostatin M Sustains the Progression Phase of Liver Regeneration through Inhibition of TGF-β2 Pathway

**DOI:** 10.3390/molecules26082231

**Published:** 2021-04-13

**Authors:** Qingjun Lu, Hao Shen, Han Yu, Jing Fu, Hui Dong, Yao Chen, Hongyang Wang

**Affiliations:** 1 State Key Laboratory of Oncogenes and Related Genes, Shanghai Cancer Institute, Renji Hospital, Shanghai Jiao Tong University School of Medicine, Shanghai 200240, China; luqjmed@alumni.sjtu.edu.cn; 2 International Cooperation Laboratory on Signal Transduction, Eastern Hepatobiliary Surgery Hospital, Second Military Medical University, Shanghai 200438, China; shenhaoehbh@smmu.edu.cn (H.S.); yuhan81@smmu.edu.cn (H.Y.); fujing@smmu.edu.cn (J.F.); donghui918@smmu.edu.cn (H.D.); 3 National Center for Liver Cancer, Second Military Medical University, Shanghai 201805, China

**Keywords:** F4/80^+^ Kupffer cells, liver regeneration, 2/3 hepatectomy, OSM, TGF-β2

## Abstract

The role of Kupffer cells (KCs) in liver regeneration is complicated and controversial. To investigate the distinct role of F4/80^+^ KCs at the different stages of the regeneration process, two-thirds partial hepatectomy (PHx) was performed in mice to induce physiological liver regeneration. In pre- or post-PHx, the clearance of KCs by intraperitoneal injection of the anti-F4/80 antibody (α-F4/80) was performed to study the distinct role of F4/80^+^ KCs during the regenerative process. In RNA sequencing of isolated F4/80^+^ KCs, the initiation phase was compared with the progression phase. Immunohistochemistry and immunofluorescence staining of Ki67, HNF-4α, CD-31, and F4/80 and Western blot of the TGF-β2 pathway were performed. Depletion of F4/80^+^ KCs in pre-PHx delayed the peak of hepatocyte proliferation from 48 h to 120 h, whereas depletion in post-PHx unexpectedly led to persistent inhibition of hepatocyte proliferation, indicating the distinct role of F4/80^+^ KCs in the initiation and progression phases of liver regeneration. F4/80^+^ KC depletion in post-PHx could significantly increase TGF-β2 serum levels, while TGF-βRI partially rescued the impaired proliferation of hepatocytes. Additionally, F4/80^+^ KC depletion in post-PHx significantly lowered the expression of oncostatin M (OSM), a key downstream mediator of interleukin-6, which is required for hepatocyte proliferation during liver regeneration. In vivo, recombinant OSM (r-OSM) treatment alleviated the inhibitory effect of α-F4/80 on the regenerative progression. Collectively, F4/80^+^ KCs release OSM to inhibit TGF-β2 activation, sustaining hepatocyte proliferation by releasing a proliferative brake.

## 1. Introduction

Liver regeneration is the most significant reaction of the liver to an injury, which is super-orchestrated by intracellular crosstalk to recover the tissue lost and to maintain homeostasis and all the hepatic functions. Despite extensive research over the last two decades, the mechanism still remains unclear. Two-thirds partial hepatectomy and the chemical injury model are the two main models used to study liver regeneration. The partial hepatectomy (PHx) model, first reported in 1932, is a traditional and reliable model used to study regeneration with a very high reproducibility [1]. The process of liver regeneration can be divided into three phases: initiation, progression, and termination [2]. It remains unclear how these phases are highly interlinked and bounded. During the initiation of regeneration, the quiescent hepatocytes are primed by cytokines to enter the cell cycle within minutes to hours after PHx. Once initiated, the hepatocytes and other hepatic cells undergo several rounds of cell cycle to enter the progression phase after PHx (approximately 8–120 h) until the residual liver is almost back to the pre-PHx liver size. Finally, regeneration is stopped to prevent liver overgrowth in the termination of liver regeneration (approximately 5–10 days after PHx), when cells exit the cell cycle and differentiate under the regulation of a series of negative cytokines [3,4].

Additionally, hepatic resection and transplantation are widely conducted among patients who suffered from liver neoplasms. Nevertheless, a large proportion of patients died of liver failure after hepatic carcinectomy or liver transplantation. The remnant liver’s ability to regenerate is very crucial for liver resection success and living donor liver transplantation. Dynamic changes occur during the regenerative process. Once initiated, hepatocytes and non-parenchymal cells in the liver undergo the progression phase until the pre-PHx liver size is basically reached. Macrophages exert significant functions in liver homeostasis, inflammation, and induction of fibrogenesis [5]. Kupffer cells (KCs), liver resident macrophages, are involved in regulating liver regeneration. However, the role of KCs in liver regeneration is still elusive and controversial. It has been reported to initiate liver regeneration through interleukin (IL)-6 and tumor necrosis factor (TNF)-α-mediated priming process, which is essential for quiescent hepatocytes to enter cell cycle [6]. CD11b^+^ KCs have been reported to promote the proliferation of hepatocytes via secreting TNF or FasL during the progression phase of liver regeneration [7]. However, KCs have also been suggested to negatively regulate the progression phase of liver regeneration by releasing inhibitory cytokines such as IL-1α/β, TGF-β, etc. [8]. F4/80 is one of the most representative surface markers of mouse monocyte macrophages, which is generally not expressed in other types of leukocytes [9]. With KCs being approximately 25% of liver mononuclear cells (MNCs) [10], it is unknown whether F4/80^+^ KCs play distinct roles during the process of liver regeneration. In our study, to distinguish KCs function between the initiation and termination phases of liver regeneration, anti-F4/80 antibody (α-F4/80) was intraperitoneally injected at different time points before or after PHx, which could clear almost 70% of F4/80^+^ KCs in mice [11,12]. Unexpectedly, selective clearance of F4/80^+^ KCs in pre- or post-PHx reveals the distinct roles during initiation and progression of liver regeneration rather than termination. RNA sequencing of isolated F4/80^+^ KCs showed very differences between the two phases. In addition to initiating regeneration, F4/80^+^ KCs sustain the progression phase of liver regeneration by releasing oncostatin M (OSM) to block TGFβ2 activation.

## 2. Results

### 2.1. F4/80^+^ KCs Play a Dual Role in Controlling the Initiation and the Progression of Liver Regeneration Rather Than the Termination

**Figure 1:**
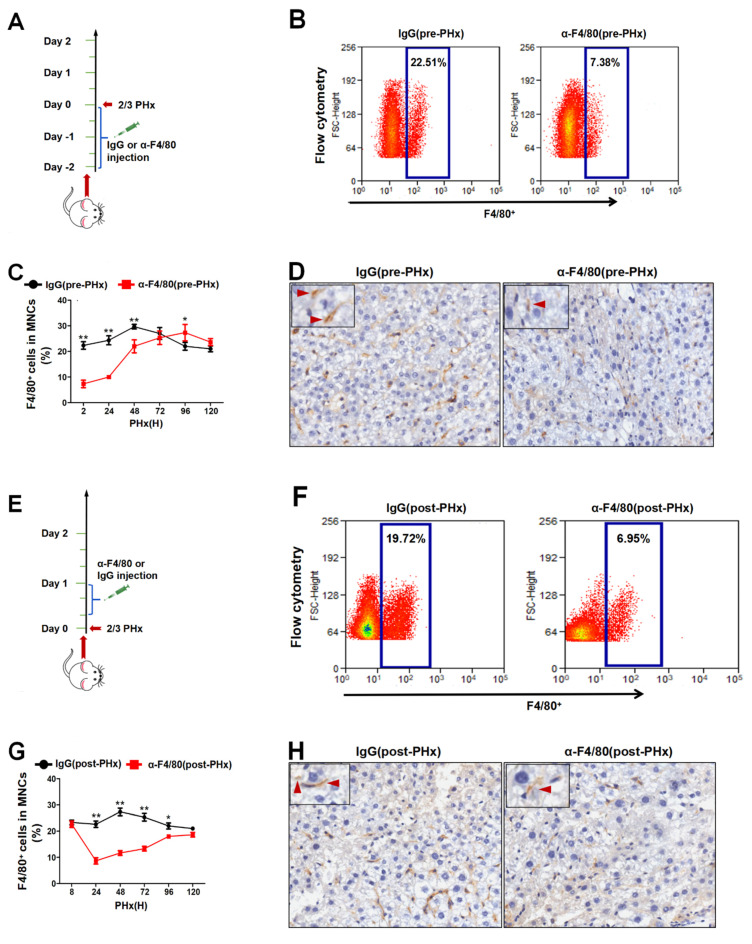
α-F4/80-mediated clearance of F4/80^+^ Kupffer cells (KCs). (**A**) Pattern graph of the depletion of KCs in pre-partial hepatectomy (PHx) α-F4/80 treatment mice. (**B**) Mice were given i.p. injections of 250 mg α-F4/80 or IgG2b at 48 h, 24 h, and 2 h before PHx. Mice were executed at different time points, and the proportion of F4/80^+^ KCs in liver mononuclear cells (MNCs) was detected by flow cytometry. (**C**) Changes in F4/80^+^ cells in the livers of pre-PHx α-F4/80 treatment mice were detected by flow cytometry. (**D**) Representative immunohistochemistry staining of F4/80^+^ cells in liver sections of pre-PHx α-F4/80 treatment mice. Red arrow represents F4/80^+^ KCs (PHx 2 h). (**E**) Pattern graph of depletion of KCs in post-PHx α-F4/80 treatment mice. (**F**) Mice were given i.p. injections of 250 mg α-F4/80 or IgG2b at 8 h, 16 h, and 24 h after PHx. Animals were executed, and the proportion of F4/80^+^ KCs in MNCs was detected by flow cytometry. (**G**) Changes in F4/80^+^ cells in the livers of post-PHx α-F4/80 treatment mice were detected by flow cytometry. (**H**) Representative immunohistochemistry staining of F4/80^+^ cells in liver sections of post-PHx α-F4/80 treatment mice. Red arrow represents F4/80^+^ KCs (PHx 24h). * *p* < 0.05, ** *p* < 0.01, *n* = 3–5.

Given the dynamic changes in macrophages in response to liver damage [13,14,15], we study whether F4/80^+^ KCs also regulate the termination of liver regeneration in addition to initiation. The injection of α-F4/80 in pre-PHx markedly eliminates almost 70% of F4/80^+^ KCs, and the elimination is sustained for 48 h after PHx (Figure 1A–D). Similar to pre-PHx intervention, the proportion of F4/80^+^ KCs also greatly decreased in post-PHx intervention (Figure 1E–H). 

**Figure 2:**
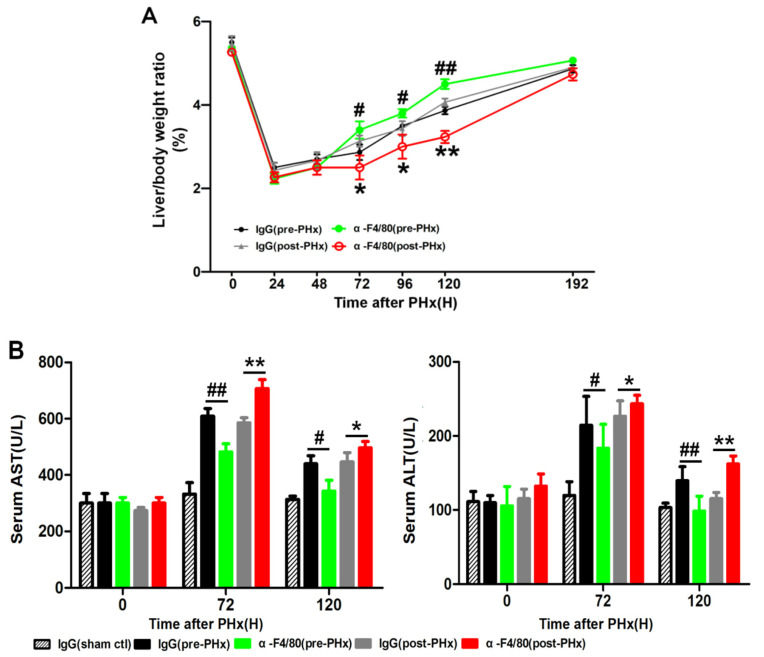

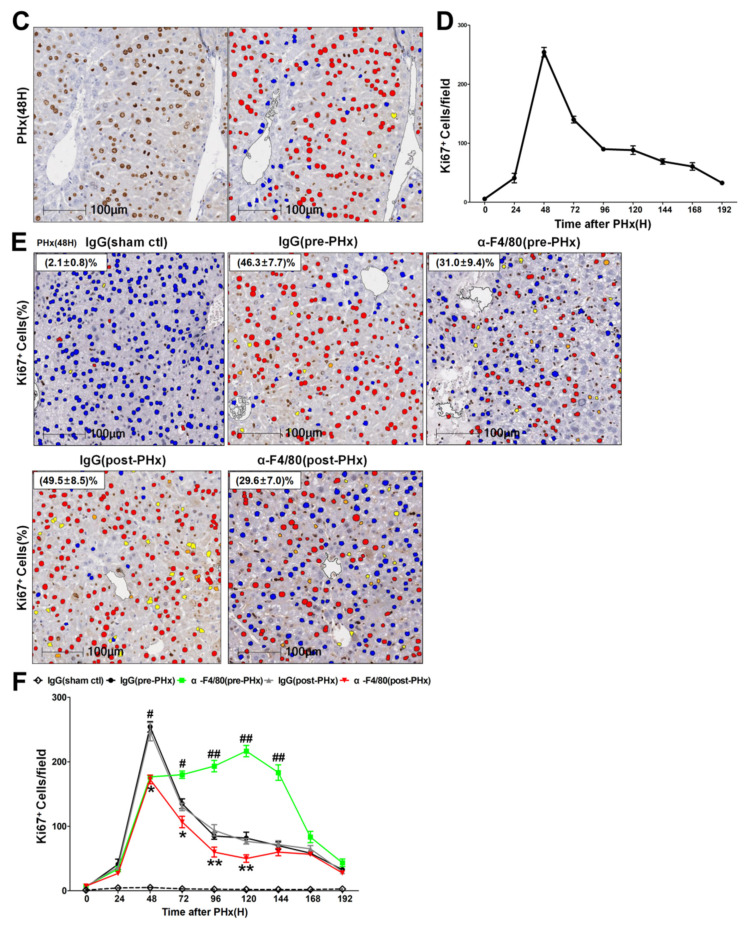
F4/80^+^ KCs play a dual role in controlling liver regeneration. (**A**) Liver/body weight ratios after PHx in IgG (pre-PHx), α-F4/80 (pre-PHx), IgG (post-PHx), and α-F4/80 (post-PHx). (**B**) Serum alanine transaminase (ALT) and serum aspartate transaminase (AST) assay from mice. (**C**) Representative immunostaining of Ki67 in liver sections obtained from mice (control) after PHx (48 h). Immunohistochemical techniques and HALO digital software were used to analyze the Ki67^+^ cells. Blue represents non-proliferating cells, yellow represents weakly positive cells, while red represents Ki67 strongly positive cells. (**D**) Quantification of the Ki67^+^ cells in liver sections obtained from mice (control) after PHx. (**E**) Representative photomicrographs of immunohistochemistry for Ki67 in the livers of pre- or post-PHx α-F4/80-treated mice. (**F**) Quantification of the Ki67^+^ cells in liver sections from IgG (sham ctl), IgG (pre-PHx), α-F4/80 (pre-PHx), IgG (post-PHx), and α-F4/80 (post-PHx). # *p* < 0.05, ## *p* < 0.01, IgG (pre-PHx) mice compared to α-F4/80 (pre-PHx) at indicated times after 2/3 PHx; * *p* < 0.05, ** *p* < 0.01, IgG (post-PHx) mice compared to α-F4/80 (post-PHx) at indicated time, *n* = 3–5.

As shown in Figure 2A, α-F4/80 post-PHx-treatment led to lower liver–body ratios than IgG-control-treatment did at 72 h, 96 h, and 120 h, especially at 120 h after PHx. At PHx day 5, the liver mass after α-F4/80 pre-PHx treatment recovered to almost 90% while that of post-PHx-treatment recovered only less than 60%. Given slightly higher serum aspartate transaminase (AST) and alanine transaminase (ALT) in the post-PHx-treated group than that in pre-PHx-treated one, F4/80^+^ KCs might affect liver resection recovery (Figure 2B). Unexpectedly, the liver mass recovered to the baseline at day 8 in both treatments. In the control group, the peak of cells proliferation (Ki67^+^ cells) appeared 48 h after PHx (Figure 2C–D). Additionally, depletion of F4/80^+^ KCs in pre-PHx significantly delayed the peak of cell proliferation from 48 to 120 h after PHx. Unexpectedly, depletion of them in post-PHx persistently lessened cell proliferation especially 2–5 days after PHx (Figure 2E–F). Together, it indicated that F4/80^+^ KCs regulated the initiation and the progression of liver regeneration but not termination.

### 2.2. F4/80+ KCs Maintain Hepatocyte Proliferation during the Progression Phase of Liver Regeneration

**Figure 3:**
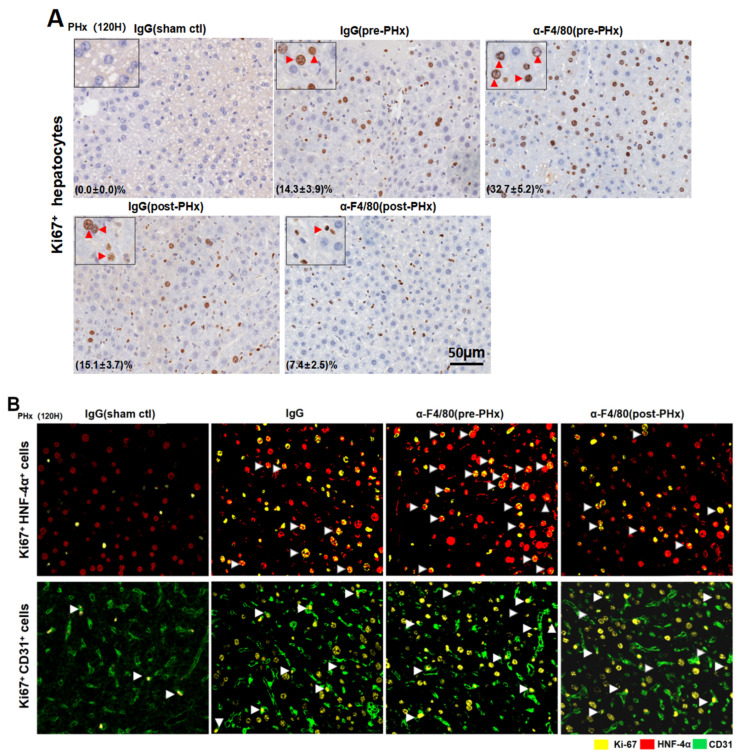

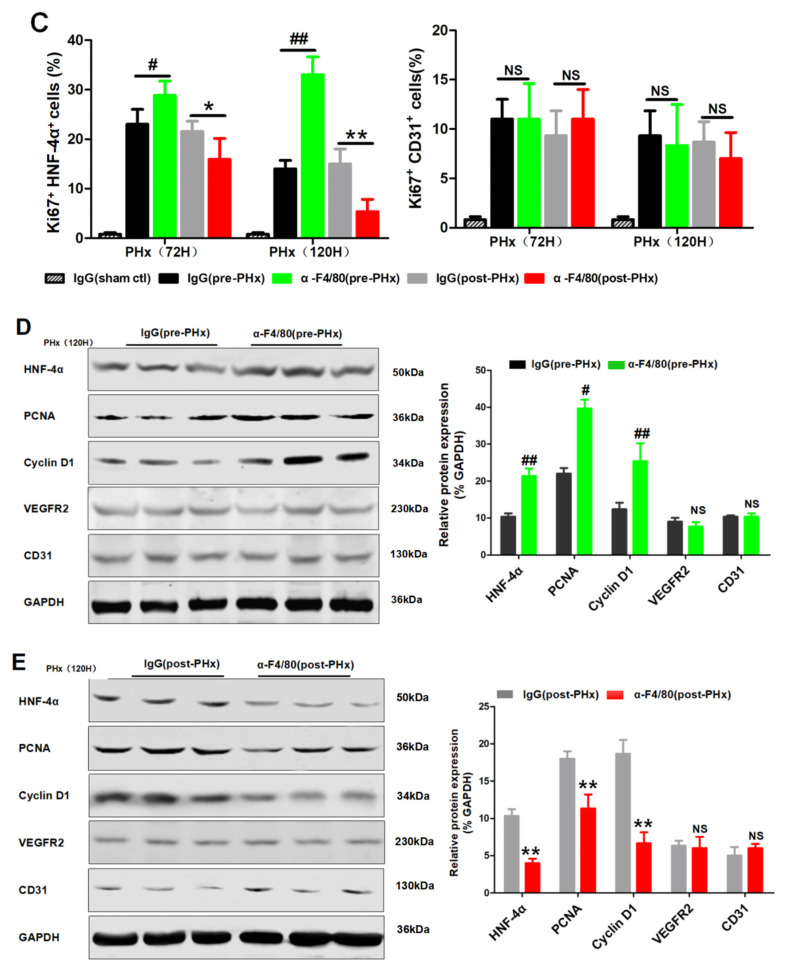
The different effects of depletion of F4/80^+^ KCs in pre- or post-PHx on liver regeneration mainly at hepatocyte proliferation. (**A**) Representative images of Ki67^+^ hepatocytes in liver tissues at 120 h after PHx. Those relatively larger and rounder (black arrow) are considered hepatocytes. Quantification of the labeled cells at the lower left corner. (**B**) Representative proliferative Ki67^+^ HNF-4α^+^ hepatocytes (white arrow) and proliferative Ki67^+^ CD31^+^ liver endothelial cells (LECs) (white arrow) in pre- or post-PHx α-F4/80-treated mice at 120 h after PHx. Hepatocytes were marked by HNF-4α (red), LECs were marked by CD31 (green), and proliferative cells were marked by Ki67 (yellow). (**C**) Quantitative analysis of Ki67^+^ HNF-4α^+^ hepatocytes and Ki67^+^ CD31^+^ LECs from (**A**). (**D**,**E**) The expression of proliferation-related proteins in hepatocytes, HNF-4α, PCNA, and Cycling D1 and in LECs, VEGFR2, and CD31 after α-F4/80 treatment in the pre-PHx or post-PHx treatment groups. GAPDH served as the internal control. # *p* < 0.05, ## *p* < 0.01, IgG (pre-PHx) mice compared to α-F4/80(pre-PHx) at indicated times after 2/3 PHx. * *p* < 0.05, ** *p* < 0.01, IgG (post-PHx) mice compared to α-F4/80 (post-PHx) at indicated time; NS, not significant, *n* = 3–5.

The liver mass recovery and physiological function after PHx mainly depends on the rapid proliferation of hepatocytes and liver endothelial cells (LECs) [16,17,18,19,20]. Confocal images showed that the depletion of F4/80^+^ KCs in pre-PHx significantly enhanced the hepatocyte proliferation (Ki67^+^/HNF-4α^+^) from 72 to 120 h after PHx. Unexpectedly, the depletion of them in post-PHx persistently lessened hepatocyte proliferation, especially 72–120 h after PHx. Contrastively, the depletion of F4/80^+^ KCs in pre- or post-PHx treatment had little effect on the number of Ki67^+^/CD31^+^ LECs (Figure 3A–C). The protein levels of HNF-4α, PCNA, and cyclin D1 were upregulated in the pre-PHx group (Figure 3D) but downregulated in the post-PHx one (Figure 3E). In contrast, no significant alteration in CD31 and VEGFR2 occurred in the pre- or post-PHx groups. These data indicated F4/80^+^ KC maintenance of hepatocyte proliferation in the progression stage of liver regeneration. 

### 2.3. Different Transcriptional Alterations Occur in F4/80^+^ KCs between the Initiation and Progression Stages of Liver Regeneration 

**Figure 4:**
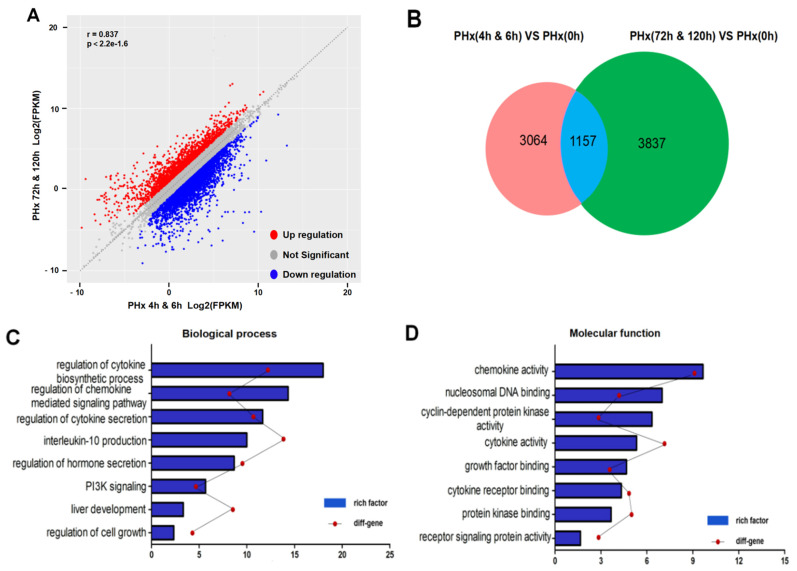
The different transcriptional alterations occur in F4/80^+^ KCs between the initiation and progression stages of liver regeneration. (**A**) RNA-seq results showed a large number of differentially expressed genes (DEGs) between the progression stage and initiation stage of liver regeneration. Red dots represent upregulated genes, while blue dots represent downregulated genes. (**B**) Venn diagram showing overlap of DEGs in PHx (72 h and 120 h) relative to PHx 0 h versus in PHx (4 h and 6 h) relative to PHx 0 h. (**C**) Enrichment of biological processes of DEGs from (**B**). (**D**) Enrichment of molecular function of DEGs from (**B**).

Transcriptional analysis of isolated KCs from hepatectomized mice at the initiation or progression phases of liver regeneration. Notably, it was quite different between the initiation (4 and 6 h after PHx) and progression phases (72 and 120 h after PHx). As is shown in Figure 4A,B, there were a large number of differentially expressed genes (DEGs) between the two phases. Gene Ontology (GO) enrichment analysis mainly focused on the cytokine biosynthetic process (Figure 4C) and chemokine activity (Figure 4D). Apparently, the constitutive differences further indicated the different functions of KCs in the initiation and progression phases of liver regeneration.

### 2.4. F4/80^+^ KCs Maintain the Progression of Liver Regeneration through Inhibition of the TGF-β2/smad2 Pathway

**Figure 5:**
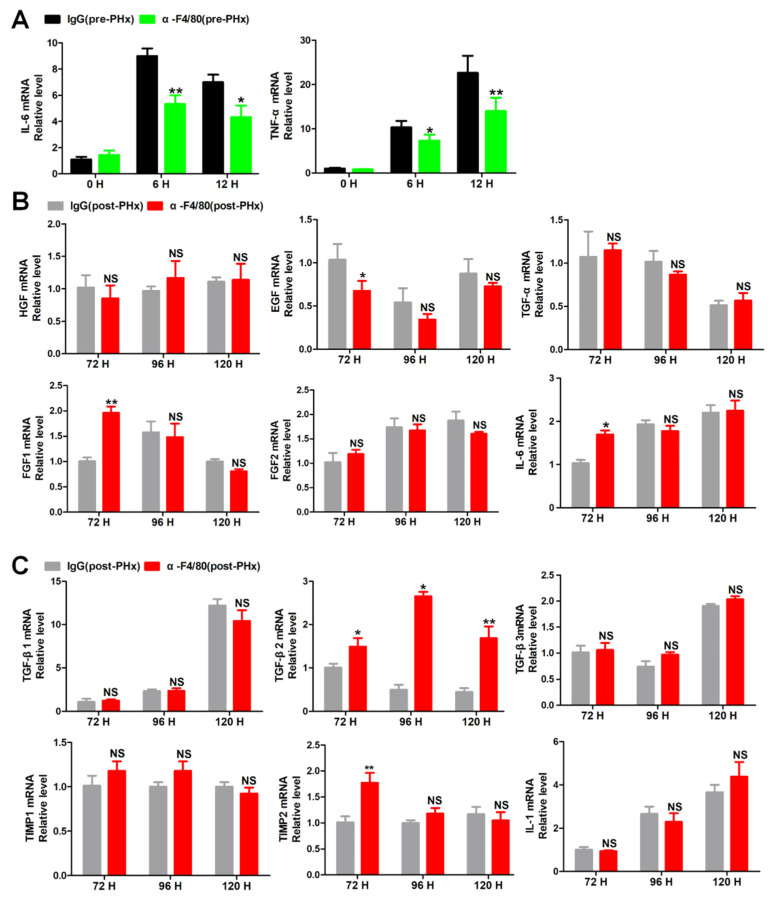
The effects of the depletion of F4/80^+^ KCs on the expression of the proliferation and anti-proliferation factors. (**A**) qRT-PCR analysis of interleukin (IL)-6 and tumor necrosis factor (TNF)-α in the livers of pre-PHx α-F4/80-treated mice. (**B**) qRT-PCR analysis of the promotion of hepatocytes mitosis-associated cytokine in the progression stage of liver regeneration. (**C**) qRT-PCR analysis of the inhibition of hepatocytes mitosis-associated cytokine in the progression stage of liver regeneration. TGF-β2 was deemed a the possible cytokine, *n* = 3–5, * *p <* 0.05, ** *p <* 0.01; NS, not significant.

**Figure 6:**
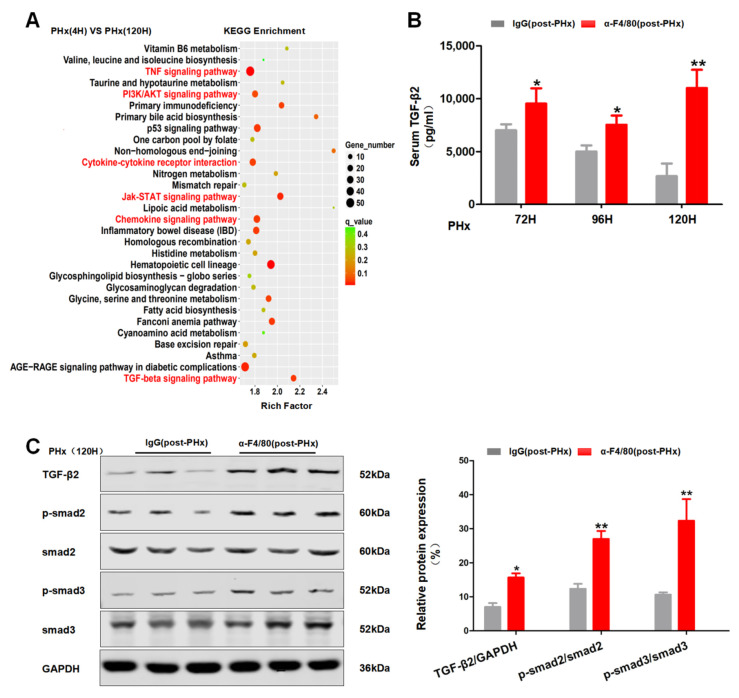
The effects of depletion of F4/80^+^ KCs post-PHx on the TGF-β2/smad2 pathway during liver regeneration. (**A**) KEGG signal pathway enrichment scatter map. (**B**) ELISA assay of serum TGF-β2 in post-PHx α-F4/80-treated mice. (**C**) Western blot of TGF-β2/smad2 pathway in livers from (**B**), *n* = 3–5; * *p <* 0.05, ** *p <* 0.01.

As shown in Figure 5A, the depletion of F4/80^+^ KCs in pre-PHx significantly decreases the expression of IL-6 and TNF-α, which are reported to play important roles in the initial of liver regeneration [14]. Thus, these data further confirmed that F4/80^+^ KCs are involved in the initial stage of liver regeneration. To further reveal the role of KCs in the progression phase of liver regeneration, quantitative real-time PCR (qRT-PCR) was analyzed for the proliferation and anti-proliferation factors [21] between IgG treatment and post-PHx F4/80^+^ KC clearance groups. Despite the fluctuation in proliferation factors between the two groups (Figure 5B), extraordinarily, the anti-proliferation factor TGF-β2 in the post-PHx F4/80^+^ KC clearance group was consistently expressed higher than that in IgG treatment group at 72 h, 96 h, and 120 h after PHx (Figure 5C). Coincidently, KEGG enrichment analysis of the isolated F4/80^+^ KCs between the two phases also implied that the TGF-β signaling pathway was involved in the progression phase of liver regeneration (Figure 6A). ELISA assays confirmed higher levels of TGF-β2 in the post-PHx group than that in the IgG treatment group (Figure 6B). In addition, phosphorylation of Smad2 or Smad3 also increased in post-PHx F4/80^+^ KC clearance (Figure 6C). Thus, TGF-β2/Smad2 pathway is possibly involved in F4/80^+^ KC-regulated progression of liver regeneration.

**Figure 7:**
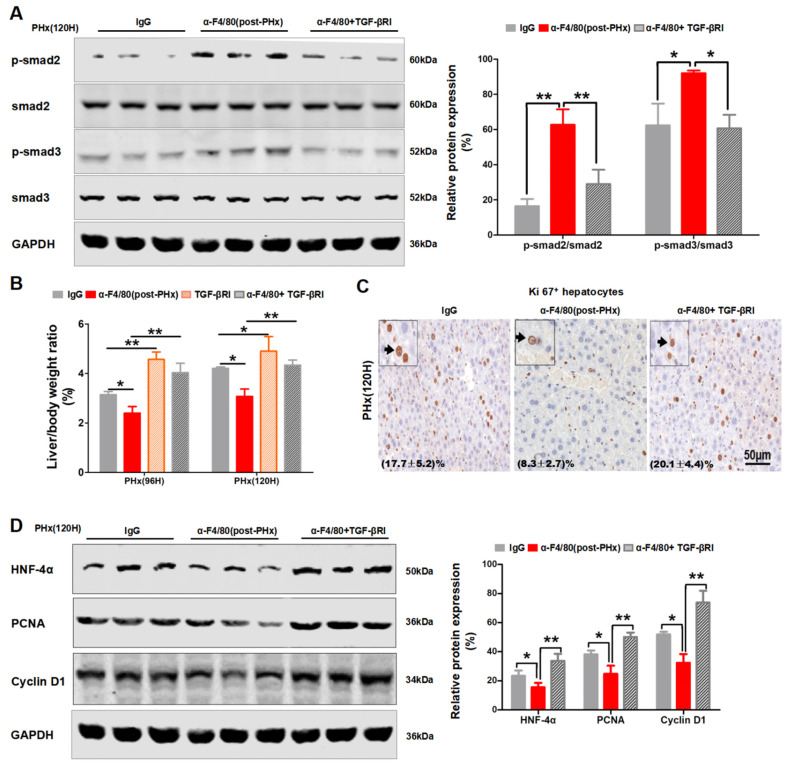
TGF-βRI treatment partially rescued the impaired proliferation of hepatocytes caused by depletion of F4/80^+^ KCs in post-PHx. (**A**) Phosphorylated and total protein levels of smad2 or smad3 in the post-PHx α-F4/80-treated groups in the absence or presence of TGF-βRI. Values represent the means ± SDs. (**B**) Liver/body weight ratios were analyzed in post-PHx α-F4/80-treated alone or that combined with TGF-βRI. (**C**) Representative photomicrographs of Ki67^+^ hepatocytes. Those relatively larger and rounder are considered hepatocytes. Quantification of the labeled cells at the lower left corner. (**D**) The protein levels of HNF-4α, PCNA, Cyclin D1, and GAPDH in livers (**A**). * *p* *<* 0.05, ** *p* *<* 0.01, *n* = 3.

To further explore this possibility, the TGF-βR inhibitor (TGF-βRI) was injected into the post-PHx α-F4/80-treated mice. As shown in Figure 7A, TGF-βRI treatment obviously inhibited the phosphorylation of smad2 and smad3 induced by post-PHx α-F4/80 injection. Moreover, TGF-βRI treatment rescued the diminishment in the liver recovery mass (Figure 7B); the number of Ki67^+^ hepatocytes (Figure 7C); and the protein levels of HNF-4α, PCNA, and Cyclin D1 (Figure 7D) in post-PHx α-F4/80-treated mice. Taken together, these data indicated that F4/80^+^ KCs sustained the progression phase of liver regeneration, likely through inhibition of the TGF-β2/smad2 pathway.

**Figure 8:**
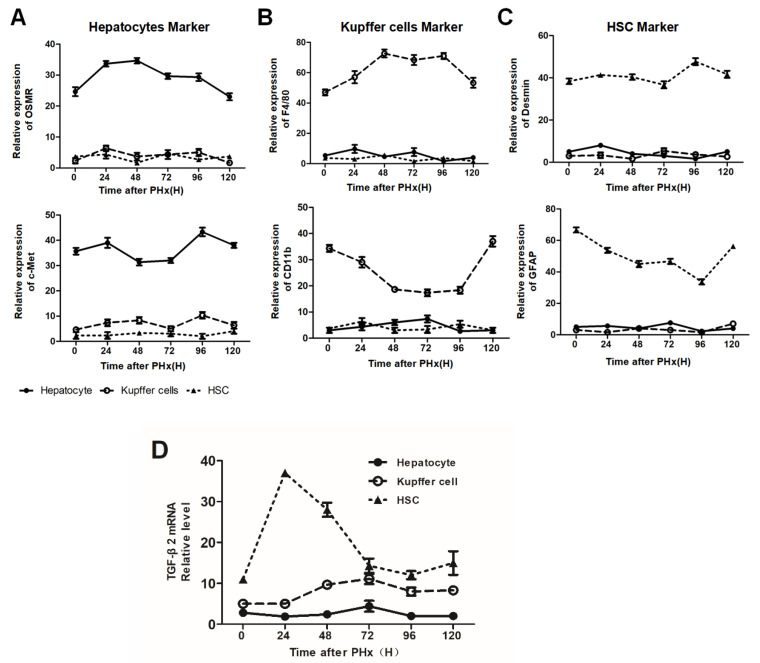
(**A**–**C**) qRT-PCR analysis of biomarkers of isolated hepatocytes, HSCs, and KCs from mice (control) after PHx. OSMR and c-Met were considered hepatocyte markers (**A**), F4/80 and CD11b were KC markers (**B**), while GFAP and Desmin were HSC markers (**C**). (**D**) Expression of TGF-β2 mRNA in primary hepatocytes, HSCs, and KCs isolated from mice (control) after PHx, *n* = 3.

In addition, we preliminarily explored the cell source of TGF-β2 in the progression phase of liver regeneration. Firstly, both parenchymal and non-parenchymal cells were isolated after PHx and detected by their biomarkers, respectively (Figure 8A–C). TGF-β2 mRNA extraordinarily fluctuated in HSCs and peaked at 24 h after PHx (Figure 8D). Combined with the TGF-β2 increase after depletion of KCs in post-PHx (Figure 6B), F4/80^+^ KCs maintain hepatocyte proliferation, possibly by affecting HSC-derived TGF-β2 during the progression phase of liver regeneration.

### 2.5. F4/80^+^ KC-Derived OSM Inhibits the TGF-β2/smad2 Pathway to Drive the Progression Phase of Liver Regeneration 

**Figure 9:**
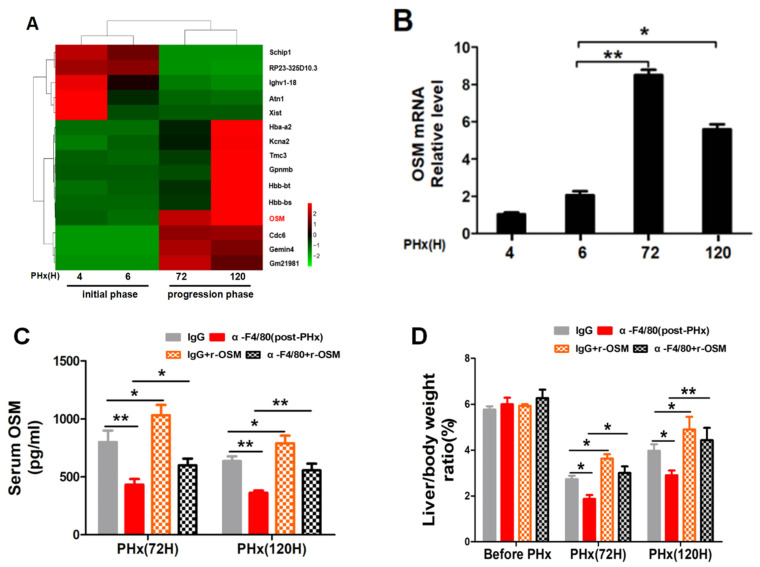
Alteration of oncostatin M (OSM) after KC depletion in post-PHx α-F4/80 treatment. (**A**) Heat map of significantly different expression genes from the initial (PHx 4 h and 6 h) or progression phase (PHx 72 h and 120 h) groups. Details of the identified genes are listed. (**B**) KCs were isolated and enriched from mice (control) at different time points after PHx, and the temporal kinetics of OSM were detected by qRT-PCR, *n* = 3. (**C**) ELISA assay of serum OSM in α-F4/80 alone or combined with r-OSM-treated mice after PHx (*n* = 3–5). (**D**) Liver/body weight ratios in mice with different treatments (**C**). * *p <* 0.05, ** *p <* 0.01, *n* = 3–5.

**Figure 10:**
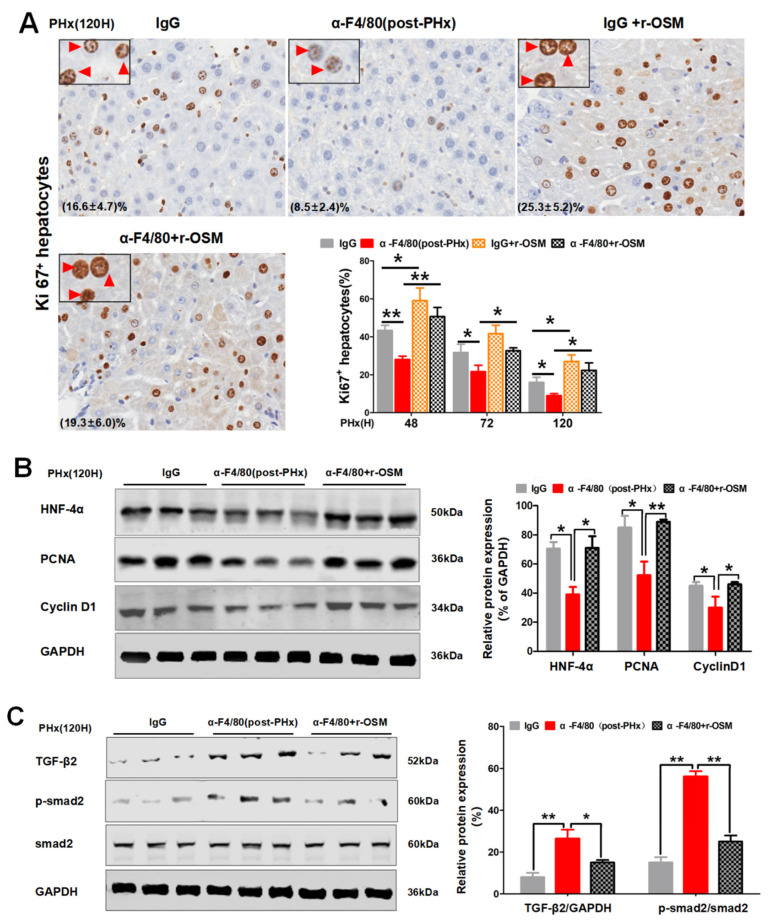
The effect of r-OSM reconstitution on hepatocyte proliferation and the TGF-β2/smad2 signaling pathway during the progression process of liver regeneration. (**A**) Representative photomicrographs and quantification of Ki67^+^ hepatocytes in α-F4/80 alone or combined with r-OSM-treated mice after PHx. (**B**) Expression of HNF-4α, PCNA, Cyclin D1, and GAPDH proteins in (**A**). (**C**) Expression of the TGF-β2/smad2 pathway-associated proteins in (**A**). *n* = 3–5, * *p* *<* 0.05, ** *p* *<* 0.01.

Analysis of the differentially expressed genes (DEGs) in isolated KCs from hepatectomized mice and the pairwise comparisons were performed between the initial (PHx 4 h and 6 h) and progression phases (PHx 72 h and 120 h). Some significantly upregulated and downregulated genes (Figure 9A) were further analyzed by the KEGG pathway and were possibly enriched in functions related to the TNF, PI3K/Akt, cytokine–cytokine receptor interaction, Jak-STAT, chemokine, and TGF-beta signaling pathways (Figure 6A). Among of them, OSM function involves these pathways [22,23,24,25,26]. Moreover, OSM acts as a regeneration-promoting cytokine to initiate liver regeneration [27,28,29]. We therefore examined the possible involvement of OSM in F4/80^+^ KCs regulating the progression phase of liver regeneration. The OSM mRNA levels at 72 or 120 h were significantly higher than that at 4 or 6 h (Figure 9B). Moreover, α-F4/80 treatment in post-PHx led to a decrease in serum OSM (Figure 9C). However, impaired liver regeneration in the post-PHx-α-F4/80 group could be rescued by the administration of r-OSM (Figure 9D); increased the number of Ki67^+^ hepatocytes (Figure 10A); and augmented the expressions of HNF-4α, PCNA, and Cyclin D1 during the progression of regeneration (Figure 10B). Moreover, α-F4/80-mediated TGF-β2 activation was significantly attenuated by r-OSM rescues in vivo (Figure 10C). These results suggested that F4/80^+^ KC-derived OSM sustained the progression phase of liver regeneration through inhibition of the TGF-β2/smad2 pathway.

## 3. Discussion

KCs serve as the liver’s immune sentry, sense diverse stimulants, and warn other hepatic cells through cytokines and cell communication [30,31]. KC activation is beneficial to liver regeneration and provides the initial priming force for hepatocyte proliferation. Hepatocytes also produce granulocyte macrophage colony stimulating factor (GM-CSF) and activate KCs during liver regeneration [14,32]. KCs promote liver regeneration after partial liver transplantation through the inhibition of apoptosis and activation of the IL-6/p-STAT3 pathway [15]. KCs initiate liver regeneration via IL-6 and TNF-α after PHx [33], and thus, the elimination of KCs delays liver regeneration and diminishes the survival of hepatectomized mice [34,35]. However, KCs have been also reported to negatively regulate liver regeneration through IL-1β, which is a negative regulator of hepatocyte proliferation [8]. Thus, the role of KCs in liver regeneration is complex and related to different experimental conditions. 

Intraperitoneal injection of α-F4/80 can remove almost 70% of F4/80^+^ KCs in mice [36]. Although the α-F4/80-eliminated efficiency of KCs is not higher than GdCl3 or liposome, it is operationally simple and fast and is able to remove F4/80^+^ KCs in different stages of liver regeneration. In our current study, intraperitoneal injection of α-F4/80 before PHx (pre-PHx) removed F4/80^+^ KCs justly in the initial phase of liver regeneration because its number recovered to the level of IgG treatment 48 h later after PHx (Figure 1C). In contrast, injection at 8 h after PHx (post-PHx) indeed eliminated them during the progression phase of regeneration (Figure 1G). Interestingly, α-F4/80 intervention at the different phases of regeneration yielded unexpected results. The latter intervention increased TGF-β2 from 72 h to 120 h after PHx, which possibly inhibited hepatocyte proliferation during the progression of liver regeneration. 

The administration of high doses of TGF-β significantly delayed DNA synthesis in rats after PHx, involved in the termination of regeneration [37]. Nevertheless, Oe et al. [38] reported that the disruption of TGF-β signaling enhanced the proliferative response of hepatocytes after PHx but did not affect the termination of liver regeneration. In our study, the administration of Ly2109761 in vivo, a selective dual inhibitor of TGF-βR I/II, indicated the involvement of the TGF-β2/Smad2 signal pathway in the progression phage of liver regeneration. TGF-β is predominately expressed in HSCs [4]. Given that the serum TGF-β2 increased in the post-PHx α-F4/80-eliminated group, we speculate that KCs communicate with HSCs to regulate the progression phase of liver regeneration. 

OSM, a member of the interleukin-6 family, shares the gp130 receptor subunit as a signal transducer and acts as a key downstream mediator of IL-6 in liver regeneration. It has been demonstrated to specifically promote differentiation of fetal hepatocytes and to control liver regeneration. OSM is mainly produced by immune cells, such as activated T lymphocytes, monocytes, and macrophages. Additionally, OSM elicits diverse biological functions in various cell types, among which the ability to regulate cell proliferation and apoptosis is most notable [38,39,40,41]. OSM decreased after α-F4/80 administration in post-PHx, while r-OSM replenishment promoted hepatocyte proliferation via inhibition of the TGF-β2/smad2 pathway, removing an endogenous growth inhibitory action. It partially explained TGF-β2 upregulation but no impact on the progression of liver regeneration.

The genome sequencing results were very different between the initiation and the progression phases during regeneration. In addition to OSM, it remains to be further studied whether other genes are involved in liver regeneration. Although KC depletion has no obvious effect on TGF-β1 expression, the role of TGF-β1 in the regeneration process could not be completely excluded because of Ly2109761 as a dual inhibitor of TGF-βRI and TGF-β-RII.

**Figure 11:**
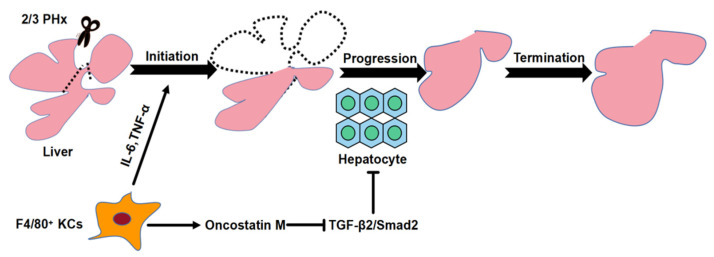
Schematic representation of the distinct roles of KCs during liver regeneration. In the process of liver regeneration, F4/80+ KCs participate in the initial stage of liver regeneration by regulating the expression of IL-6 and TNF-α. Besides, F4/80+ KCs sustain the progression phase of liver regeneration by releasing OSM to block TGFβ2 activation.

Collectively, F4/80^+^ KCs are involved not only in initiating liver regeneration but also in maintaining progression (Figure 11). KC-derived OSM inhibits TGF-β2 to sustain hepatocyte proliferation during the progression phase of liver regeneration.

## 4. Methods

### 4.1. Animal Treatments and 2/3 PHx

All experiments were conducted in accordance with the Guide for the Care and Use of Laboratory Animals published by the National Institutes of Health; 6–8-week old male mice were subjected to standard 70% PHx under chloral hydrate anesthesia [1].

F4/80^+^ KCs were specifically depleted by i.p. injection of InVivoMAb anti-mouse F4/80 antibodies (α-F4/80, BioXcell, West Lebanon, NH, USA). For pre-PHx α-F4/80 treatment, α-F4/80 or control isotype antibodies (IgG2b, BioXcell) were diluted in PBS. The mice were intraperitoneally injected with 250 mg α-F4/80 or IgG2b at 48 h, 24 h, and 2 h before PHx. For post-PHx α-F4/80 treatment, the mice were intraperitoneally injected with 250 mg α-F4/80 or IgG2b at 8 h, 16 h, and 24 h after PHx. Finally, the liver tissues collected between 0 and 192 h postoperatively were removed, weighed, and normalized to body weight.

For TGF-βR inhibitor (LY2109761, Cat#M2081, Abmole, Houston, TX, USA) treatments, TGF-βRI was diluted in PBS. On the basis of post-PHx α-F4/80 treatment, mice were given intragastric administration (50 mg/kg, p.o.) of TGF-βRI at 24 h after PHx.

For r-OSM treatments, r-OSM(Cat#50112-M08H, Sino Biological, Beijing, China) was diluted in PBS. On the basis of post-PHx α-F4/80 treatment, mice were given i.p. injection of r-OSM at 24 h after PHx.

### 4.2. Isolation of Primary Hepatocytes and Nonparenchymal Cells (NPCs)

Primary hepatocytes and NPCs were isolated from mice. Briefly, mice were anaesthetized by 5 mL/Kg-5% chloral hydrate and their portal veins were cannulated with detaining needle; then, the inferior vena cava was cut and the liver was perfused at 5 mL/min through the inferior vena cava with D-Hanks (B430, basalmedia, Shanghai, China) with 0.5 mM EDTA and 0.5% BSA at 37 °C for 5 min; D-Hanks (B410, basalmedia) containing 0.5% collagen-IV (C5138, Sigma, St Louis, MO, USA) was applied for digestion of the liver; and after perfusion for an appropriate time, the liver was dissociated in a suspension buffer and filtered with a 100 μm pore cell strainer and collected by centrifugation at 50× *g* for 5 min. Hepatocytes were pelleted and enriched using a percoll cushion (45%) and pelleted. The supernatant containing NPCs were pelleted by centrifugation at 300× *g* for 10 min.

### 4.3. Library Construction for RNA-Seq and Sequencing Procedures

For RNA-seq, F4/80^+^ KCs were isolated from NPCs by staining with APC-conjugated anti-F4/80 Ab (Cat#123116, BioLgend, San Diego, CA, USA) by flow cytometry at different time points (4, 6, 72, and 120 h after PHx). Flow cytometry was carried out with a MoFlo™ XDP Cell Sorter (BECKMAN Coulter, Miami, FL, USA). Data analysis was conducted using Summit 5.2 software (Beckman, Miami, FL, USA). The total RNA of KCs was isolated using RNeasy mini kit (Qiagen, Valencia, CA, USA). Strand-specific libraries were prepared using the TruSeq Stranded Total RNA Sample Preparation kit (Illumina, San Diego, CA, USA) following the manufacturer’s instructions. Briefly, mRNA was enriched with oligo(dT) beads. Following purification, the mRNA was fragmented into small pieces using divalent cations under 94 °C for 8 min. The cleaved RNA fragments were copied into first-strand cDNA using reverse transcriptase and random primers. This was followed by second-strand cDNA synthesis using DNA polymerase I and RNase H. These cDNA fragments then went through an end repair process, the addition of a single “A” base, and then ligation of the adapters. The products were then purified and enriched with PCR to create the final cDNA library. The purified libraries were quantified by Qubit 2.0 Fluorometer (Life Technologies, Carlsbad, CA, USA) and validated by Agilent 2100 bioanalyzer (Agilent Technologies, Palo Alto, CA, USA) to confirm the insert size and to calculate the mole concentration. A cluster was generated by cBot with the library diluted to 10 pM and then was sequenced on the Illumina HiSeq X ten (Illumina, San Diego, CA, USA). The library construction and sequencing were performed at Shanghai Biotechnology Corporation. The Differentially Expressed Genes (DEGs) in KCs of different stages of liver regeneration and the related functional clusters were compared and analyzed.

### 4.4. Immunofluorescence and Immunohistochemical (IHC) Staining

The liver samples were fixed in 10% buffered formalin overnight, dehydrated, and embedded in paraffin; 5 μm paraffin sections of the liver tissue samples were used for immunofluorescence and IHC staining.

For immunofluorescence microscopy, the liver sections were blocked (1% BSA/10% goat serum) and incubated in primary antibodies: F4/80 (1:100, Abcam, Cambridge, MA, USA), Ki67 (1:100, abcam), HNF4α (1:1000, abcam), and CD31 (1:50, abcam). The immunofluorescence was amplified by the tyramide signal amplification (TSA) systems (PerkinElmer, Waltham, MA, USA). After incubation with the primary antibody, HRP-conjugated second antibodies and then TSA plus fluorescein (NEL701A001KT, PerkinElmer) were added to the section. Another primary antibody was incubated in the same section and followed by second antibody and TSA plus Tetramethyliver regenerationhodamine (NEL702001KT, PerkinElmer). A third primary antibody and TSA plus Cyanine 5 (NEL705A001KT, PerkinElmer) were then added. Images were captured on confocal microscopy (Leica BMI-6000, Cambridge, UK); for every slide, ten representative fields were captured randomly and analyzed by Image-Pro Plus v6.0 software.

The immunohistochemistry procedures were as follows. Paraffin sections were incubated with the following antibodies: Ki67 (1:100, Abcam, Cambridge, MA, USA), and anti-rabbit or anti-mouse peroxidase-conjugated secondary antibodies (1:50, Santa Cruz Biotechnology, Santa Cruz, CA, USA) were applied, and the DAB Substrate (Dako, Hamburg, Germany) was used for coloration. Counterstaining was performed with hematoxylin (Sigma, St Louis, MO, USA). Photographs of the three representative fields were captured randomly by the Image-Pro Plus v6.0 software (Media Cybernetics Inc, Bethesda, MD, USA), and analysis of the digital images were accomplished using Image-Pro Plus v6.0 software.

### 4.5. Digital Image Analysis of Ki67 with Virtual Dual Staining

HALO platform version 3.1.1076 (Indica Labs, Corrales, NM, USA) was used for digital image analysis of Ki67. Furthermore, hepatocytes were distinguished by setting the nuclear contrast threshold, minimum nuclear OD, nuclear size, and minimum nuclear roundness.

### 4.6. Real-Time PCR

The trizol method was used to isolate total RNA from liver tissues or cell lysates according to the manufacturer’s instructions (Gibco, Carlsbad, CA, USA). qRT-PCR was performed using a SYBR Green Premix Ex Taq (Takara Bio inc., Otsu, Shiga, Japan) on Light Cycler^®^ 96 (Roche, Basel, Switzerland). qRT-PCR data were normalized to actin. Furthermore, qRT-PCR analysis of the biomarkers of hepatocytes (c-Met and OSMR), HSCs (GFAP and Desmin), or KCs (F4/80 and CD11b) isolated from mice was conducted. The primer sequences are shown in Table 1.

### 4.7. Western Blotting

The liver tissues were lysed in a RIPA buffer, and total protein concentration was determined using the BCA protein assay kit (Pierce/Thermo Scientific, Rockford, IL, USA). Sixty micrograms of protein were separated by SDS-PAGE and transferred onto nitrocellulose membranes (Osmonics, Minnetonka, MN, USA). After blocking with 0.5% BSA, the membranes were incubated with primary antibodies at 4 °C overnight and then with secondary antibodies for at least 2 h. The primary antibodies used were as follows: TGF-β2 (1:1000, R&D Systems, Wiesbaden, Germany); CyclinD1 (1:1000, proteintech, Wuhan, China); PCNA (1:2000, proteintech, Wuhan, China); VEGFR2 (1:1000, Cell Signaling Technology, Danvers, MA, USA); HNF-4α, GAPDH, and CD31 (1:500, Abcam, Cambridge, MA, USA); and VEGFR2, Smad2, Smad3, and phospho-Smad2/3 (1:1000, Cell Signaling Technology, Danvers, MA, USA). The secondary antibodies were obtained from Invitrogen (1:5000, Thermo Fisher Scientific, Waltham, MA, USA). Finally, protein bands were determined using Odyssey Infrared Imaging system (Odyssey Classic, LI-COR Biosciences, Lincoln, NE, USA), and its density was quantified using Image Studio Lite Ver 3.1 (v5.2, LI-COR Biosciences, Lincoln, NE, USA). The density of protein bands were normalized against GAPDH.

### 4.8. Measurement of the Serum ALT, AST, TGF-β2, and OSM

Blood samples were withdrawn from the inferior vena cava of mice under anesthesia. The serum levels of aspartate transaminase (AST) and alanine transaminase (ALT) were determined by an automatic biochemical analyzer (MGC 240, Thermo Scientific™ Indiko™). In addition, the serum levels of TGF-β2 (CUSABIO Biotech, Wuhan, China) and OSM (CUSABIO Biotech, Wuhan, China) were measured by ELISA Kits according to the manufacturer’s protocols.

### 4.9. Statistical Analysis

Statistical analysis was carried out using GraphPad Prism 5 software (GraphPad Software, La Jolla, CA, USA). Data are presented as means ± standard deviations (SD). Statistical significance was calculated using Student’s *t* test. *p* < 0.05 was considered significant. Means ± SDs are shown in Figures where applicable.

## Figures and Tables

**Table 1:** List of the primer sequences.

Gene Name	Forward Primer	Reverse Primer
F4/80	CTGCACCTGTAAACGAGGCTT	GCAGACTGAGTTAGGACCACAA
CD11b	CCATGACCTTCCAAGAGAATGC	ACCGGCTTGTGCTGTAGTC
GFAP	CGGAGACGCATCACCTCTG	TGGAGGAGTCATTCGAGACAA
Desmin	CCTGGAGCGCAGAATCGAAT	TGAGTCAAGTCTGAAACCTTGGA
β-actin	GGCTGTATTCCCCTCCATCG	CCAGTTGGTAACAATGCCATGT
HGF	AACAGGGGCTTTACGTTCACT	CGTCCCTTTATAGCTGCCTCC
TNF-α	CTGAACTTCGGGGTGATCGG	GGCTTGTCACTCGAATTTTGAGA
IL-6	CTGCAAGAGACTTCCATCCAG	AGTGGTATAGACAGGTCTGTTGG
EGF	AGAGCATCTCTCGGATTGACC	CCCGTTAAGGAAAACTCTTAGCA
TGF-α	CACTCTGGGTACGTGGGTG	CACAGGTGATAATGAGGACAGC
TGF-β1	CTTCAATACGTCAGACATTCGGG	GTAACGCCAGGAATTGTTGCTA
TGF-β2	TCGACATGGATCAGTTTATGCG	CCCTGGTACTGTTGTAGATGGA
OSM	CCCGGCACAATATCCTCGG	TCTGGTGTTGTAGTGGACCGT
FGF1	GGGGAGATCACAACCTTCGC	GTCCCTTGTCCCATCCACG
TGF-β3	GGACTTCGGCCACATCAAGAA	TAGGGGACGTGGGTCATCAC
FGF2	GCGACCCACACGTCAAACTA	CCGTCCATCTTCCTTCATAGC
PEDF	CCAACTTCGGCTACGATCTGT	TCTGTTCGATGTTCAGCTCCC
TIMP-1	CGAGACCACCTTATACCAGCG	ATGACTGGGGTGTAGGCGTA
TIMP-2	TCAGAGCCAAAGCAGTGAGC	GCCGTGTAGATAAACTCGATGTC
Met	GTGAACATGAAGTATCAGCTCCC	TGTAGTTTGTGGCTCCGAGAT

## Data Availability

Any data can be available from the first author.

## Sample Availability

Samples of the compounds are available from the authors.
